# 1A6/DRIM, a Novel t-UTP, Activates RNA Polymerase I Transcription and Promotes Cell Proliferation

**DOI:** 10.1371/journal.pone.0014244

**Published:** 2010-12-07

**Authors:** Qunhui Peng, Jianguo Wu, Ying Zhang, Yun Liu, Ruirui Kong, Lelin Hu, Xiaojuan Du, Yang Ke

**Affiliations:** 1 Key Laboratory of Carcinogenesis and Translational Research (Ministry of Education), Genetics Laboratory, Peking University School of Oncology, Beijing Cancer Hospital & Institute, Beijing, China; 2 Department of Cell Biology, Peking University Health Science Center, Beijing, China; 3 Cancer Research Center, Peking University Health Science Center, Beijing, China; Texas A&M University, United States of America

## Abstract

**Background:**

Ribosome biogenesis is required for protein synthesis and cell proliferation. Ribosome subunits are assembled in the nucleolus following transcription of a 47S ribosome RNA precursor by RNA polymerase I and rRNA processing to produce mature 18S, 28S and 5.8S rRNAs. The 18S rRNA is incorporated into the ribosomal small subunit, whereas the 28S and 5.8S rRNAs are incorporated into the ribosomal large subunit. Pol I transcription and rRNA processing are coordinated processes and this coordination has been demonstrated to be mediated by a subset of U3 proteins known as t-UTPs. Up to date, five t-UTPs have been identified in humans but the mechanism(s) that function in the t-UTP(s) activation of Pol I remain unknown. In this study we have identified 1A6/DRIM, which was identified as UTP20 in our previous study, as a t-UTP. In the present study, we investigated the function and mechanism of 1A6/DRIM in Pol I transcription.

**Methodology/Principal Findings:**

Knockdown of 1A6/DRIM by siRNA resulted in a decreased 47S pre-rRNA level as determined by Northern blotting. Ectopic expression of 1A6/DRIM activated and knockdown of 1A6/DRIM inhibited the human rDNA promoter as evaluated with luciferase reporter. Chromatin immunoprecipitation (ChIP) experiments showed that 1A6/DRIM bound UBF and the rDNA promoter. Re-ChIP assay showed that 1A6/DRIM interacts with UBF at the rDNA promoter. Immunoprecipitation confirmed the interaction between 1A6/DRIM and the nucleolar acetyl-transferase hALP. It is of note that knockdown of 1A6/DRIM dramatically inhibited UBF acetylation. A finding of significance was that 1A6/DRIM depletion, as a kind of nucleolar stress, caused an increase in p53 level and inhibited cell proliferation by arresting cells at G1.

**Conclusions:**

We identify 1A6/DRIM as a novel t-UTP. Our results suggest that 1A6/DRIM activates Pol I transcription most likely by associating with both hALP and UBF and thereby affecting the acetylation of UBF.

## Introduction

In eukaryotes, the nucleolus is a compartment for ribosome biosynthesis which includes transcription of ribosomal RNA precursor (pre-rRNA), processing of pre-rRNA, and assembly of ribosomal subunits. The ribosomal gene (rDNA) is first transcribed by RNA polymerase I (Pol I) to produce a 47S pre-rRNA containing the sequences for 5′- external transcribed spacer (5′-ETS), 18S rRNA, internal transcribed spacer-1 (ITS1), 5.8S rRNA, internal transcribed spacer-2 (ITS2), 28S rRNA and 3′- external transcribed spacer (3′-ETS). After chemical modification at numerous sites, the 47S pre-rRNA is processed to produce 18S rRNA, 5.8S rRNA, and 28S rRNA. The 18S rRNA is incorporated into the ribosomal small subunit, whereas the 28S and 5.8S rRNAs are incorporated into the ribosomal large subunit.

In humans, transcription by Pol I requires the upstream binding factor (UBF), and the TBP-containing promoter selectivity factor SL-1 in addition to RNA Pol I [1], [2]. UBF is a high mobility group (HMG) box sequence-specific DNA-binding protein, which binds to the rDNA promoter and recruits SL1 [3], [4], [5]. SL1 is a species-specific complex which includes TBP, TAF_II_48, TAF_II_ 63 and TAF_II_110 and is essential for reconstitution of Pol I transcription [6], [7], [8]. As a key component in Pol I transcription, UBF activity is tightly regulated by association with transcriptional factors and itself undergoing posttranslational modifications. Under different cell growth conditions, the activity of UBF is controlled mainly by phosphorylation and acetylation. UBF is phosphorylated at multiple sites in growing cells [9], [10], but is hypophosphorylated and transcriptionally inactive in quiescent cells [11], [12]. Acetylation of UBF also differs during cell cycle progression in accordance with its functioning in the control of rDNA transcription. UBF is acetylated in S and G2 phase and is deacetylated in mitosis and early G1 [13]. For acetylation of UBF, CBP and Rb-HDAC are key regulators which function in a “flip-flop” manner [14]. It has been found that acetylation and deacetylation regulate UBF activity without affecting its DNA binding properties. Instead, UBF acetylation activates Pol I transcription by enhancing the association between UBF and Pol I components [15].

Pol I transcription and pre-rRNA processing are believed to be coordinated in plants, yeast and mammalian cells [16], [17]. This coordination takes place in a “terminal knob” that is visible under electron microscopy which is a large 90S pre-ribosome complex known as ribosomal small subunit (SSU) processome [18], [19], [20], [21]. This SSU processome contains 12S U3 snoRNP, MPP10 complex, t-UTPs, bUTP, BMS/RCL1 complex, RNA helicases and RNA-binding proteins [22]. The coordination between Pol I transcription and rRNA processing is mediated by t-UTPs which are required for both 18S rRNA processing and Pol I transcription, and in addition, are associated with rDNA [23]. A nucleolar protein may be identified as a classical UTP if it is associated with U3 snoRNA and its depletion results in inhibition of 18S rRNA processing but does not affect Pol I transcription. Up to now, only seven UTPs have been identified as t-UTPs in yeast including UTP4, UTP5, UTP8, UTP9, UTP10, UTP15 and UTP17 [23]. Human orthologs of yeast UTP4, UTP5, UTP10, UTP15 and UTP17 have been identified as t-UTPs, but BLAST searches have failed to identify orthologs of yeast UTP8 and UTP9 in humans [24]. However, the mechanisms by which these t-UTPs function in Pol I transcription remain undetermined.

Our previous study identified 1A6/DRIM as the human UTP20 which functions mainly in the processing of 18S rRNA [25]. Proteomics studies have found that yeast UTP20 (YBL004w) resides in the 90S preribosome in the nucleolus [26], [27]. However, whether 1A6/DRIM is a classical UTP or a t-UTP remains unknown. In the present study, we investigated the function of 1A6/DRIM in Pol I transcription and found that 1A6/DRIM functions as a t-UTP. Further study demonstrated that 1A6/DRIM affected UBF acetylation and raises the possibility that there is a novel mechanism by which t-UTPs activate Pol I transcription via altering modification of UBF or other Pol I transcription preinitiation complex factors.

## Results

### Knockdown of 1A6/DRIM results in a decrease of 47S pre-rRNA

**Figure 1:**
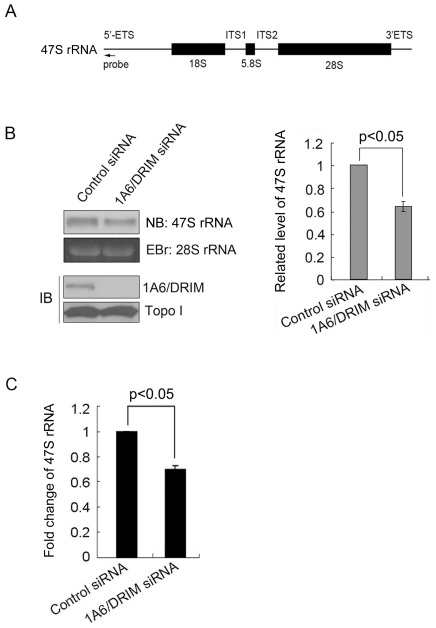
Knockdown of 1A6/DRIM results in a decrease in 47S rRNA. A. Cartoon illustrating the location of the probe on the 47S rRNA used for Northern blotting to detect 47S rRNA transcript. B. A 1A6/DRIM specific siRNA (si2-8) was transfected into U2OS cells. RNA was extracted 72 hours posttransfection, resolved on a 1% glyoxal-agarose gel and transferred onto a positively charged nylon membrane. Blots were probed with a Biotin-labeled DNA oligonucleotide (as shown in A) to detect 47S rRNA (left upper panel). Ethidium Bromide (EB) staining of the 28S rRNA on the agarose gel was used as a loading control. Cell lysates were prepared 72 hours after siRNA transfection and proteins from the lysates were separated on SDS-PAGE and transferred onto a PVDF membrane. Blots were probed with anti-1A6/DRIM antibody. Topoisomerase I (Topo I) was used as a loading control (left lower). The experiment was repeated three times and the difference in the densitometry scanning of the 47S rRNA bands is displayed in the right panel. C. A 1A6/DRIM specific siRNA (si2-8) was transfected into U2OS cells. RNA was extracted 72 hours posttransfection and reverse transcription was carried out. Real time PCR was performed with primers amplifying the 5′-extreme end fragment of the 47S rRNA. The experiment was repeated three times in duplicate and the statistical significance of the difference is shown. Statistical analyses were performed with two-tailed unpaired *t* test.

The 47S pre-rRNA is transcribed by Pol I and the extreme 5′ end of the external transcribed sequence of the 47S pre-rRNA is rapidly processed in the cell, so it was used for Northern blotting to determine the rate of Pol I transcription initiation [28]. To determine whether 1A6/DRIM functions in Pol I transcription, 47S rRNA level was evaluated with Northern blotting when 1A6/DRIM was silenced by siRNA. The oligonucleotide used as a probe for Northern blotting for detecting 47S rRNA was described previously [29] and is illustrated in Figure 1A. Figure 1B shows that the 47S rRNA level was decreased in 1A6/DRIM deficient cells (left panel). This experiment was repeated three times and the difference in the densitometry scanning of the 47S rRNA bands is shown in Figure 1B (right panel). This result suggested that knockdown of 1A6/DRIM considerably inhibited 47S rRNA transcription. Inhibition of 47S rRNA transcription by knockdown of 1A6/DRIM was confirmed by real time PCR. Reverse transcription was carried out with total RNA extracted from U2OS cells after 1A6/DRIM was silenced. Real time PCR was performed with primers amplifying the extreme 5′ end of the external transcribed sequence of the pre-rRNA. As shown in Figure 1C, the level of 47S rRNA significantly decreased in 1A6/DRIM depleted cells as compared with the control siRNA treated cells.

### 1A6/DRIM activated the rDNA promoter luciferase reporter

**Figure 2:**
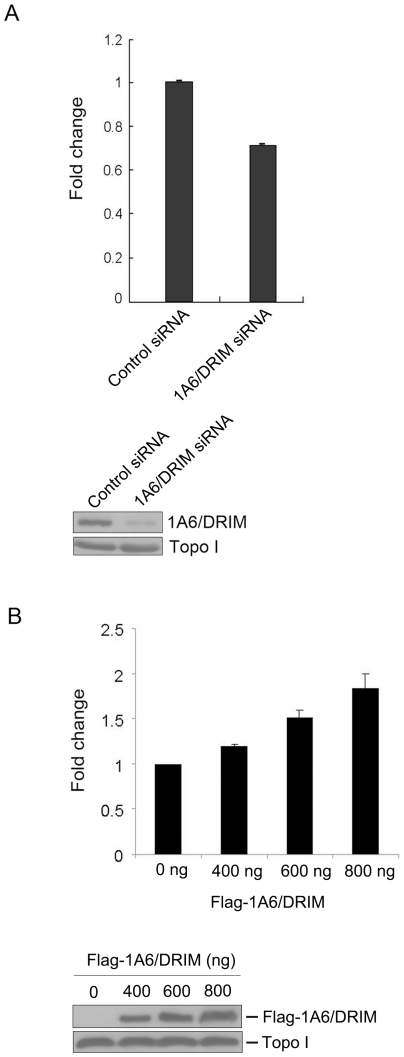
1A6/DRIM activates rDNA promoter luciferase reporter. A. A 1A6/DRIM specific siRNA (si2-8) was transfected into U2OS cells. pHrD-IRES-Luc plasmid was transfected into the cells 48 hours after transfection of siRNA. Luciferase activity was measured 24 hours after transfection of pHrD-IRES-Luc. Results which are summarized from three independent repetitions of the experiments are shown (upper panel). Proteins from the above transfection were subjected to Western blotting for detection of 1A6/DRIM (lower panel). Topoisomerase I (Topo I) was used as a loading control. B. pHrD-IRES-Luc plasmid was cotransfected with increasing amounts of pCI-neo-Flag-1A6/DRIM plasmid. Luciferase activity was measured 24 hours after transfection. Results are summarized from three independent experiments in duplicate (upper panel). Cell lysates were prepared 24 hours after transfection and proteins from cell lystaes were separated by SDS-PAGE and transferred onto a PVDF membrane. The upper part of the blot was probed with anti-Flag antibody M2 and the lower part was probed with anti-topoisomerase I (Topo I) as a loading control.

The human rRNA promoter luciferase reporter plasmid pHrD-IRES-Luc has been demonstrated to reflect Pol I transcription activity [30]. To further confirm the effect of 1A6/DRIM on Pol I transcription, pHrD-IRES-Luc was constructed as described previously [30] and was transfected into U2OS cells 48 hours after transfection with 1A6/DRIM-specific siRNA. Luciferase activity was determined 24 hours after transfection with pHrD-IRES-Luc. Figure 2A shows that knockdown of 1A6/DRIM inhibited the activity of pHrD-IRES-Luc. In addition, plasmid coding Flag-1A6/DRIM was cotransfected with pHrD-IRES-Luc into U2OS cells and luciferase activity was measured 24 hours posttransfection. As shown in Figure 2B, 1A6/DRIM activated the pHrD-IRES-Luc reporter in a dose-dependent manner. Taken together, these results demonstrated that 1A6/DRIM activates Pol I transcription.

### 1A6/DRIM bound the rDNA promoter and interacted with UBF at the promoter

**Figure 3:**
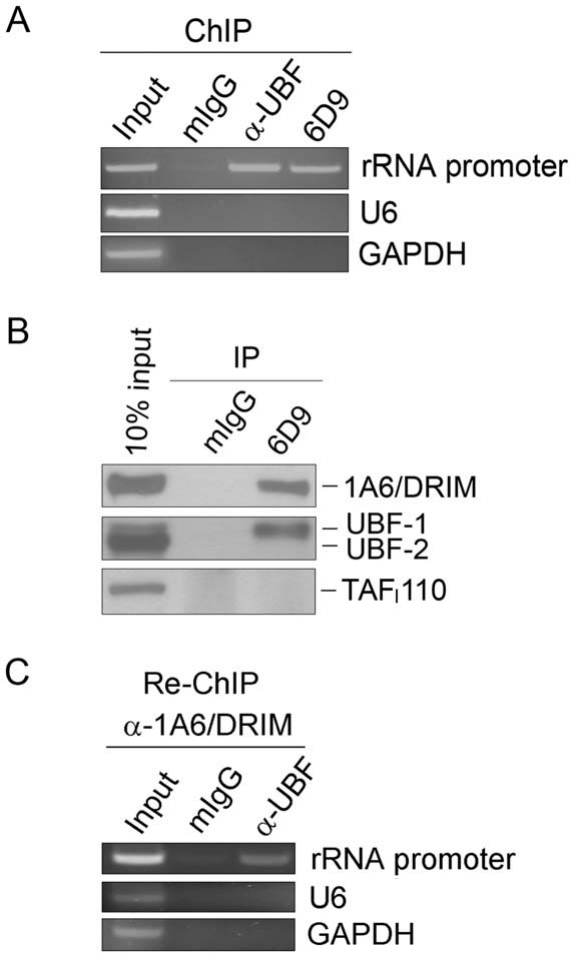
1A6/DRIM bound rDNA promoter and interacted with UBF at the rDNA promoter. A. Chromatin immunoprecipitation (ChIP) was performed with antibodies directed against either 1A6/DRIM or UBF. Mouse IgG (mIgG) was used as an antibody control. A DNA fragment of the rDNA promoter sequence was amplified by PCR. DNA fragments from either the U6 or GAPDH promoter were amplified as controls. The PCR products were resolved on a 2% agarose gel and stained with EB. B. Whole cell lysate was extracted from U2OS cells. Immunoprecipitation was carried out with anti-1A6/DRIM antibody. Proteins from 1A6/DRIM specific immunoprecipitates were separated on SDS-PAGE and blotted onto PVDF membranes. The upper part of the blot was probed with anti-1A6/DRIM antibody and the lower part of the same blot was hybridized with anti-UBF antibody or anti-TAF_I_110 antibody. C. Re-ChIP experiment was performed to confirm that 1A6/DRIM bound UBF at the rDNA promoter. ChIP was performed using an anti-1A6/DRIM antibody. Re-ChIP was carried out using either mouse IgG (mIgG) or an anti-UBF antibody. A DNA fragment of the rDNA promoter sequence was amplified by PCR. DNA fragments from either the U6 or GAPDH promoter were amplified as controls. The PCR products were resolved on a 2% agarose gel and stained with EB.

To determine whether 1A6/DRIM binds rDNA in cells, chromatin immunoprecipitation experiments (ChIP) were performed with anti-1A6/DRIM antibody. A DNA fragment from the rDNA promoter was amplified by PCR with DNA extracted from 1A6/DRIM specific immunoprecipitates. Figure 3A shows 1A6/DRIM bound rDNA promoter. In this experiment, UBF which is known to be an rDNA binding transcription factor was used as a positive control.

To determine whether 1A6/DRIM is associated with UBF, immunoprecipitation was performed with anti-1A6/DRIM antibody and UBF was evaluated by Western blotting on 1A6/DRIM-specific immunoprecipitates. Figure 3B shows that UBF was present in the 1A6/DRIM-specific immunocomplex, demonstrating that 1A6/DRIM was associated with UBF. We also examined TAF_I_ 110 from 1A6/DRIM-specific immunoprecipitates. As shown in Figure 3B, TAF_I_ 110 was not found in the 1A6/DRIM-specific immunocomplex, indicating that 1A6/DRIM specifically interacts with UBF. UBF has two isoforms including the 97kD UBF-1 and the 94kD UBF-2 [31]. UBF-1 is known as the constitutively active form of UBF, whereas UBF-2 is responsible for tuning the response of ribosomal RNA genes to growth factor stimulation [32]. It is notable that UBF-1 was enriched in the 1A6/DRIM immunoprecipitates, suggesting 1A6/DRIM may affect UBF activity by specifically interacting with UBF-1. To demonstrate that 1A6/DRIM and UBF form a complex on rDNA, a re-ChIP experiment was performed. A ChIP assay was first performed with 1A6/DRIM-specific antibody, and the precipitates were subjected to re-ChIP assay with UBF antibody. As shown in Figure 3C, the rDNA promoter sequence was amplified from the re-ChIP precipitate, demonstrating that 1A6/DRIM bound UBF at the rDNA promoter.

### 1A6/DRIM is associated with hALP and knockdown of 1A6/DRIM inhibits UBF acetylation

**Figure 4:**
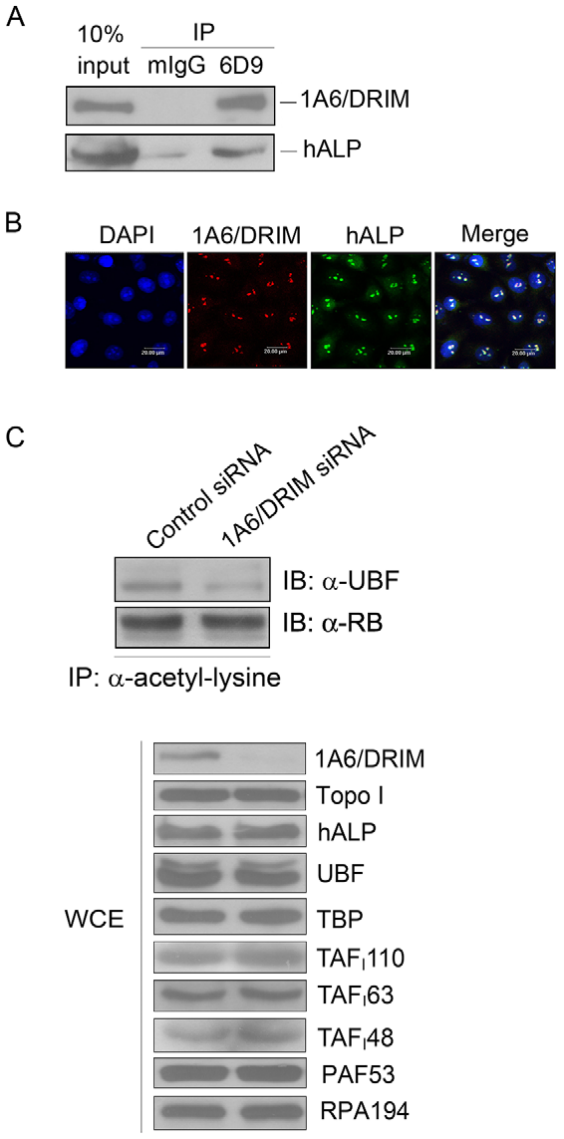
1A6/DRIM was associated with hALP and knockdown of 1A6/DRIM inhibited UBF acetylation. A. Immunoprecipitation was performed with the 1A6/DRIM-specific monoclonal antibody 6D9. Proteins from the immunoprecipitates were separated by SDA-PAGE and transferred onto a PVDF membrane. The upper part of the blot was hybridized with 1A6/DRIM antibody and the lower part was probed with anti-hALP antibody. B. Indirect immunofluorescence staining was performed with a monoclonal antibody directed against 1A6/DRIM and a hALP-specific polyclonal antibody. 1A6/DRIM specific immunocomplexes were detected with TRITC-conjugated anti-mouse IgG and hALP specific immunocomplexes were detected with FITC-conjugated anti-rabbit IgG. Images were photographed under confocal microscopy after the nucleus was stained with DAPI. C. A 1A6/DRIM specific siRNA (si2-8) was transfected into U2OS cells. Cell lysate was prepared 72 hours posttransfection. Immunoprecipitation was performed with anti-acetyl-lysine on the cell lysates. Proteins from the immunoprecipitates were separated on SDS-PAGE and transferred onto a PVDF membrane. Blots were probed with anti-UBF antibody or anti-RB antibody (upper panel). Proteins from the whole cell lysates (WCE) were directly subjected to Western blotting to detect protein levels of indicated proteins (lower panel). Topoisomerase I (Topo I) was used as a loading control.

To determine the mechanisms by which 1A6/DRIM functions in Pol I transcription, we isolated 1A6/DRIM-interacting proteins using the yeast two-hybrid assay. A nucleolar acetyl-transferase hALP was found to interact with 1A6/DRIM, and we found that hALP activated Pol I transcription by acetylating UBF (manuscript submitted). We then performed immunoprecipitation with 1A6/DRIM specific antibody 6D9 to confirm the interaction between 1A6/DRIM and hALP. As shown in Figure 4A, hALP indeed interacted with 1A6/DRIM. In addition, cellular localization of 1A6/DRIM and hALP was analyzed with indirect immunofluorescence. Figure 4B shows that 1A6/DRIM colocalized with hALP in the nucleolus, further confirming the interaction between 1A6/DRIM and hALP. We next sought to determine whether 1A6/DRIM affects UBF acetylation. Immunoprecipitation was performed with anti-acetyl-lysine antibody on cell lysates extracted from 1A6/DRIM depleted U2OS cells or a control siRNA treated cells. Proteins from the immunoprecipitates were subjected to Western blotting for detection of hALP. Figure 4C shows that UBF acetylation was dramatically inhibited in 1A6/DRIM depleted cells and the total protein level of UBF was not changed by 1A6/DRIM knockdown (Figure 4C, lower panel). We asked if knockdown of 1A6/DRIM specifically affects acetylation of UBF. To address this issue, acetylation of RB was evaluated after 1A6/DRIM was depleted. Immunoprecipitation was performed with anti-acetyl-lysine antibody on cell lysates extracted from 1A6/DRIM depleted U2OS cells or a control siRNA treated cells. Proteins from the immunoprecipitates were subjected to Western blotting for detection of RB. As shown in Figure 4C (upper panel), acetylation of RB was not altered by depletion of 1A6/DRIM, demonstrating that 1A6/DRIM specifically affects acetylation of UBF. It was also shown that depletion of 1A6/DRIM had no effects on protein levels of hALP or on levels of Pol I subunit RPA194 and the Pol I associated factor 53 (PAF53). In addition, protein levels of TBP and SL1 complex components including TAF_I_110, TAF_I_63 and TAF_I_48 were not affected by knockdown of 1A6/DRIM (Figure 4C, lower panel).

### Knockdown of 1A6/DRIM causes p53 activation and inhibits cell proliferation by arresting cells at G1

**Figure 5:**
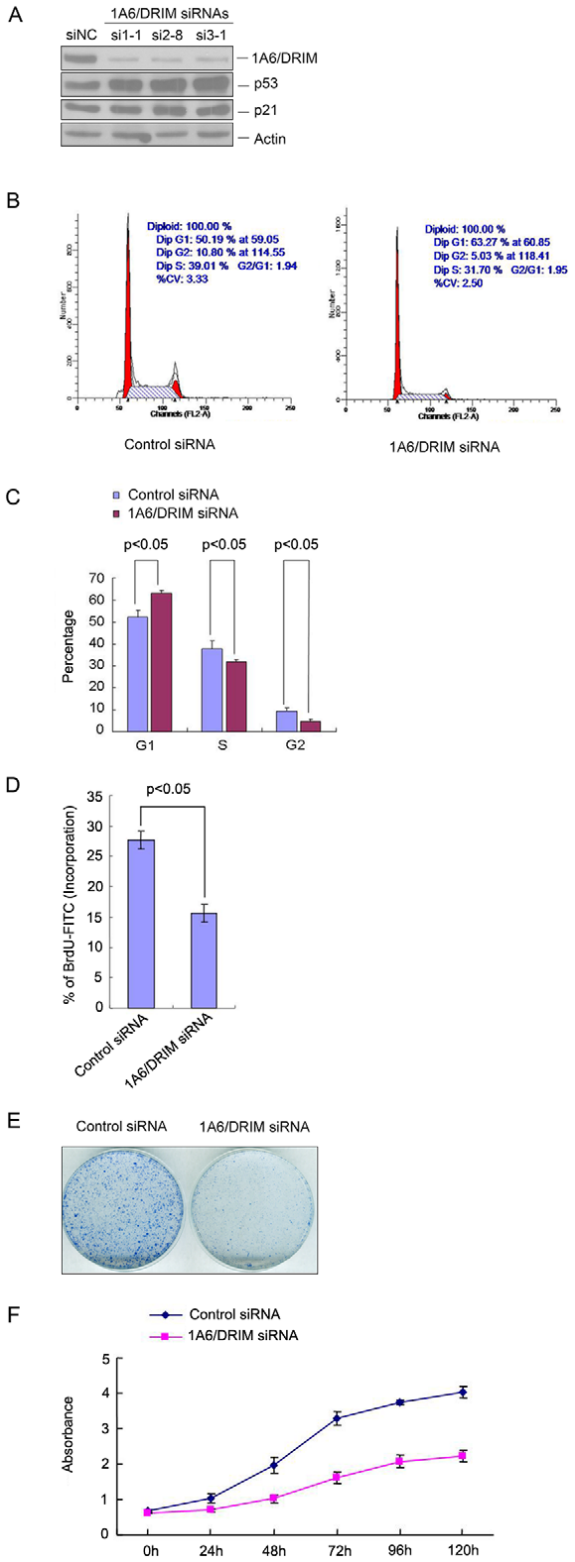
Knockdown of 1A6/DRIM activated p53 and inhibited cell proliferation by arresting cells at G1. A. U2OS cells were transfected with three 1A6/DRIM siRNAs (si1-1, si2-8 and si3-1) and whole cell extracts were prepared 72 hours posttransfection. Proteins from the extracts were separated on SDS-PAGE, and transferred onto a PVDF membrane. Blots were probed with antibodies directed against 1A6/DRIM, p53 or p21. Beta-actin was used as a loading control. B and C. U2OS cells were transfected with a 1A6/DRIM siRNA (si2-8). The cell cycle was analyzed with flow cytometry 72 hours posttransfection. A representative result is shown in B and a summary of results from three independent experiments is shown in C. D. U2OS cells were transfected with a 1A6/DRIM siRNA (si2-8). Cells were pulsed for 16 hours with BrdU, and were then stained with FITC-conjugated anti-BrdU and 7-AAD. BrdU and 7-AAD staining were analyzed by FACScan (Becton Dickinson). S-phase population was plotted by comparing FITC (DNA incorporation) vs. 7-AAD (total DNA) staining. Shown is the data summarized from three independent experiments in duplicate. E.U2OS cells were transfected with a 1A6/DRIM siRNA (si2-8). Two thousand cells were seeded in 6 mm plates 72 hours posttransfection. Cells were grown for 10 days and cell colonies were stained with Coomassie Bright Blue after fixation with ethanol/acetic acid. F. U2OS cells were transfected with a 1A6/DRIM siRNA (si2-8). Cells were seeded in 96-well plates 72 hours posttransfection. Ten microliters of CCK-8 was added to the cells and absorbance at 450 nm was measured at different time periods. The experiment was repeated three times in duplicate and growth curves were plotted using absorbance at 450 nm vs. different time points. Statistics analysis was performed with two-tailed unpaired *t*-test in C and D.

It has been found that disruption of nucleolar function activates p53 [33]. The level of p53 protein increased when 1A6/DRIM was silenced in U2OS cells, as shown in Figure 5A, and in addition, p21 was activated in 1A6/DRIM depleted cells. To study the biological significance of p53 activation resulting from 1A6/DRIM depletion, the cell cycle was analyzed after 1A6/DRIM silencing in U2OS cells. A representative result is shown in Figure 5B that knockdown of 1A6/DRIM caused an increased cell numbers in G1 and decreased cell numbers in S and G2. This experiment was repeated three times in duplicate and the data is summarized in Figure 5C. The results showed that there were 63.3% of the cells in G1, 32.1% in S and 4.6% in G2 in 1A6/DRIM depleted cells, while there were 51.6% of the cells in G1, 38.4% in S and 10.0% in G2 in a control siRNA treated cells suggesting knockdown of 1A6/DRIM caused arrest of cells at G1 (p<0.05). To confirm this observation, the same experiment was carried out in MCF-7 cells and identical results were obtained (data not shown). In addition, the BrdU incorporation assay was performed to evaluate cell numbers in S phase when 1A6/DRIM was silenced by siRNA. As shown in Figure 5D the result showed that knockdown of 1A6/DRIM resulted in significantly fewer cells in S phase confirming that knockdown of 1A6/DRIM caused cell arrest at G1. Accordingly, cell proliferation was examined with colony formation. Figure 5E shows that both of the size and the number of colonies decreased when 1A6/DRIM was silenced. To quantify the effect of 1A6/DRIM depletion on cell proliferation, a cell growth curve was plotted using the Cell Count Kit-8 assay. As shown in Figure 5F, growth of 1A6/DRIM depleted cells was significantly inhibited compared to control siRNA treated cells. Thus, we demonstrated that knockdown of 1A6/DRIM inhibited cell proliferation by arresting cells at G1.

## Discussion

Pol I transcription and rRNA processing are coordinated and stringently controlled in normal cells. This coordination is mediated by the interaction of UBF with pre-rRNA processing factors [17]. The SSU processome has been found to play a role in the coordination of Pol I transcription and pre-rRNA processing. The SSU processome is assembled on nascent pre-rRNA as a large complex with a multimodular structure [34]. While Pol I transcription is undergoing elongation, the SSU processome participates simultaneously in pre-rRNA processing. Pre-rRNA is sequentially cleaved at A0, A1 and A2. Upon cleavage at the A2 site, the pre-40S particle containing major components of the SSU processome and 18S rRNA departs from the remaining pre-rRNA on which another subset of ribosomal proteins are recruited to form the pre-60S particle [34]. It is known that t-UTPs are required for Pol I transcription and early stages of pre-rRNA processing. Up to now, only seven t-UTPs have been identified in yeast and five t-UTPs have been identified in mammalian cells. However, the mechanisms by which t-UTPs function in Pol I transcription remain unknown.

Our previous study identified 1A6/DRIM as the human UTP20 that functions mainly in 18S rRNA processing [25]. The extreme 5′ end of the external transcribed sequence of the pre-rRNA is rapidly processed in the cell, so it has been used in Northern blotting to determine the rate of Pol I transcription initiation [28]. In the present study, we evaluated the level of 47S rRNA by using the extreme 5′ end of the external transcribed sequence of the pre-rRNA as a probe, and the results showed that silencing of 1A6/DRIM results in a decreased level of 47S pre-rRNA, indicating that knockdown of 1A6/DRIM inhibited Pol I transcription. Since the rRNA promoter is transcribed by Pol I, the human rRNA promoter luciferase reporter plasmid pHrD-IRES-Luc has been demonstrated to reflect Pol I transcription activity [30]. We found that 1A6/DRIM expression activated rDNA promoter luciferase reporter activity and knockdown of 1A6/DRIM inhibited this activity, further demonstrating that 1A6/DRIM activates Pol I transcription. In addition, 1A6/DRIM is associated with the rDNA promoter. We therefore can identify 1A6/DRIM as a novel t-UTP. Interestingly, we showed that 1A6/DRIM specifically interacts with UBF-1, the constitutively active form of UBF, suggesting the interaction between 1A6/DRIM and UBF may play an important role in rDNA transcription activation.

t-UTPs functions in a complex to activate Pol I transcription, and Pol I transcription is regulated mainly by phosphorylation and acetylation of UBF. We therefore speculated that 1A6/DRIM might also interact with kinases or acetyl-transferases in addition to binding with UBF. In our previous study we found several 1A6/DRIM-interacting proteins by using the yeast two-hybrid assay, among which was the nucleolar acetyl-transferase, hALP. hALP is a nucleolar protein with multiple functions in cell division related to its capacity for acetylating histone and tubulin [35], [36]. This prompted study of the function of hALP in Pol I transcription and we found hALP activates Pol I transcription by acetylating UBF (manuscript submitted). In the present study, the interaction between 1A6/DRIM and hALP was verified by immunoprecipitation and immunofluorescence staining. Importantly, knockdown of 1A6/DRIM dramatically inhibited UBF acetylation, suggesting that 1A6/DRIM may affect UBF acetylation by forming and maintaining an acetyl-transferase-containing complex with UBF. Thus, we provide a mechanism by which t-UTPs activates Pol I transcription. We propose that other t-UTPs may function in Pol I transcription via formation of complexes with kinases, phosphotases or other acetyl-transferases to regulate phosphorylation or acetylation and therefore regulate the activity of UBF.

Nucleolar functional disruptions have been shown to generate nucleolar stress signaling to p53 [33]. As expected, we found that knockdown of 1A6/DRIM also activated p53. Defects in ribosome biogenesis cause p53 activation via a L11-dependent pathway [37]. However, how knockdown of 1A6/DRIM mediates p53 activation requires further investigation. In the present study, activation of p53 was verified by an elevated level of p21. Significantly, knockdown of 1A6/DRIM arrested cells at G1. Consistent with this result, 1A6/DRIM depletion inhibited cell proliferation. Up to this point, we have demonstrated that 1A6/DRIM is a novel t-UTP which activates Pol I transcription at least partly by regulating UBF modification. UBF is deacetylated in mitosis and early G1 and is acetylated in S and G2 phase [13]. We propose that 1A6/DRIM may facilitate acetylation of UBF at late G1 and therefore promotes cell cycle progression from G1 to S phase. In addition, cell growth is controlled by protein synthesis which is dependent on ribosome biogenesis. Thus, we suggest that 1A6/DRIM promotes cell proliferation via its function in activation of rRNA transcription by Pol I and subsequent rRNA processing.

Ribosome biogenesis factors play crucial roles in cell proliferation, and deregulation of these factors, such as Pes1 which functions in 28S rRNA processing, often causes tumorigenesis [38], [39]. Moreover, 1A6/DRIM has been found to be upregulated in some tumors (our unpublished data) and we are therefore currently investigating the 1A6/DRIM expression profile in human tumors to determine whether 1A6/DRIM can be used as a diagnostic marker or a therapeutic target in human tumors.

## Materials and Methods

### Cell culture and 1A6/DRIM RNAi

U2OS and MCF-7 cells were obtained from the American Type Culture Collection (ATCC) and were grown according to the instructions provided by the ATCC. Cells were incubated in a humidified atmosphere with 5% CO_2_ at 37°C. For silencing 1A6/DRIM expression, three siRNAs targeting 1A6/DRIM were chemically synthesized as follows: si1-1: 5′- GACAAAGCCCGUUUCCCAC-3′; si2-8: 5′-GCCAUAGCCUGAAAGAUUU-3′; si3-1: 5′- GGCAAAGUUGUUCUGUCUU-3′; together with synthesis of an unrelated siRNA (siNC: 5′-ACUACCGUUGUUAUAGGUG-3′) for a control (Shanghai GenePharma Co., Ltd). The synthesized siRNA was transfected into cells at a concentration of 100 nM with Lipofectamine 2000™ (Invitrogen) according to the manufacturer's instructions.

### Plasmids

A plasmid coding Flag-1A6/DRIM was generated by inserting the 1A6/DRIM cDNA fragment obtained by RT-PCR into pCI-neo-Flag. A human rDNA promoter-luciferase reporter pHrD-IRES-Luc was constructed as previously described [30].

### Preparation of cellular extracts and immunoprecipitation

Immunoprecipitation was performed as described previously [40]. Briefly, U2OS cell lysates were prepared in NET buffer (150 mM NaCl, 5 mM EDTA, 50 mM Tris-Cl [pH7.5]) supplemented with 1% NP-40 and protein cocktail inhibitor. Cell lysates were incubated with anti-1A6/DRIM anbibody 6D9 or mouse IgG crosslinked protein A sepharose beads (Amersham Biosciences) for 4 h at 4°C. After washing twice with NET+1% NP-40 and twice with NET buffer, the precipitated proteins were subjected to Western blotting.

### Immunoblotting

Proteins were separated on SDS-PAGE and transferred onto PVDF membranes (Amersham Biosciences). Blots were probed sequentially with corresponding antibodies and HRP-conjugated secondary antibodies. Immunocomplexes were detected with ECL Western blot Detection Reagent (GE Healthcare) before exposure to X-ray film.

### ChIP assay

Chromatin immunoprecipitation (ChIP) was performed as described previously [41]. Briefly, cells were fixed with 1% formaldehyde. Immunoprecipitation was performed with antibodies coupled to protein A/G-Sepharose. Immunoprecipitated chromatin-derived DNA was analyzed by PCR with primers specific for the rDNA promoter as described previously [42] (forward primer: 5′-CGCTGCTCCCGCGTGTGTCC-3′; reverse primer: 5′-CAGCGACAGGTCGCCAGAGG-3′). The primer sequences used to amplify U6 and GAPDH promoters were as described previously [43]. PCR products were resolved on agarose gel and visualized with ethidium bromide.

### Northern blotting and RT-PCR

For detection of 47S rRNA, RNA was resolved on a 1% glyoxal-agarose gel (NorthernMax®-Gly kit, Ambion) and blotted onto BrightStar®-PLUS positively charged Nylon membranes (Ambion). DNA oligonucleotide was biotin-labeled with a Biotin 3′ End DNA Labeling Kit (PIERCE) to probe for 47S rRNA and the sequence of the oligonucleotide which was employed was as described previously [29]: 5′- CCTCTCCAGCGACAGGTCGCCAGAGGACAGCGTGTCAGCAATAACCCGGCGGCCAAAATG-3′. Hybridization was performed as described previously [44] and hybridized 47S rRNA was detected with the BrightStar® BioDetect™ Nonisotopic Detection Kit (Ambion). Reverse transcription was performed with total RNAs using SuperScript™ II Reverse Transcriptase (Invitrogen) according to the manufacturer's instructions. Real-time PCR for amplification of the 47S rRNA was performed with the following primers: Forward (+180 to +196): 5′-CGGGTCCGGGTCTCTGA-3′; Reverse (+307 to +326): 5′-GGAGACGAGAACGCCTGACA-3′.

### Immunofluorescence

Cells were plated on coverslips in 6-well plates one day before harvest. Double indirect immunofluorescence was performed with the monoclonal anti-1A6/DRIM antibody 6D9 and a polyclonal antibody directed against hALP after cells were fixed. 1A6/DRIM specific immunocomplexes were detected with TRITC-conjugated goat anti-mouse IgG and hALP specific immunocomplexes were detected with FITC-conjugated goat anti-rabbit IgG. Immunofluorescence signals were recorded by confocal laser scanning microscopy (Leica TCS-ST2).

### Flow cytometry analysis

For DNA content analysis, cells were trypsinized and fixed in 75% ice-cold ethanol at 4°C overnight. Following RNase digestion, cells were stained with propidium iodide (PI). Flow cytometry analysis was performed evaluating red (PI) emission (at 630 nm). Data from 10^4^ cells were collected and analyzed by using Cellquest software (Becton Dickinson).

### BrdU incorporation assay

The cell population in S-phase was determined with a BD Pharmingen™ BrdU Flow Kit according to the manufacturer's instructions. Briefly, cells were pulsed for 16 hours with bromodeoxyuridine (BrdU), and were then stained with fluorescein isothiocyanate (FITC)-conjugated anti-BrdU and 7-amino-actinomycin D (7-AAD). Positive BrdU staining and 7-AAD staining were analyzed by FACScan (Becton Dickinson). Data from 10^4^ cells were analyzed by using Cellquest software (Becton Dickinson). Cells in S-phase were quantitated by comparing FITC (DNA incorporation) vs. 7-AAD (total DNA) staining.

### Growth curve

Growth curves were plotted with a Cell Counting Kit-8 (CCK-8, Dojindo) according to the manufacturer's instructions. In brief, cells were seeded in 96-well plates. CCK-8 [2-(2-methoxy-4-nitrophenyl)-3-(4-nitrophenyl)-5-(2,4-disulfophenyl)-2H-tetrazolium, monosodium salt] was added to the cells and incubated for 4 hours. Absorbance at 450 nm was measured using a microplate reader. The experiment was repeated three times in duplicate. Growth curves were plotted using the mean ± SD of absorbance at 450 nm vs. time points.
